# Did the COVID-19 pandemic change the weight reduction in patients with obesity after bariatric surgery?

**DOI:** 10.1186/s12889-023-16837-8

**Published:** 2023-10-11

**Authors:** Somayeh Mokhber, Ali Sheidaei, Shahrzad Ahmadkaraji, Seyed Amin Setarehdan, Seyed Mohsen Rahimi, Fatemeh Sadat Hosseini-Baharanchi, Ali Mazaherinezhad, Abdolreza Pazouki

**Affiliations:** 1 Minimally Invasive Surgery Research Center, Iran University of Medical Sciences, Mansouri St., Niyayesh St., Sattarkhan Ave., Rasool-E-Akram Hospital, Tehran, Iran; 2 Department of Epidemiology and Biostatistics, School of Public Health, Tehran University of Medical Sciences, Tehran, Iran; 3 Department of Sports and Exercise Medicine, School of Medicine, Iran University of Medical Sciences, Tehran, Iran; 4 Department of Biostatistics, School of Public Health, Iran University of Medical Sciences, Tehran, Iran; 5 Center of Excellence for Minimally Invasive Surgery Training, Iran University of Medical Sciences, Tehran, Iran; 6 Center of Excellence of European Branch of International Federation for Surgery of Obesity, Tehran, Iran

**Keywords:** COVID-19, Weight loss, One anastomose gastric bypass, Roux-en-Y gastric bypass, Sleeve gastrectomy

## Abstract

**Background:**

The COVID-19 pandemic has significantly impacted healthcare services worldwide, including bariatric surgery. There is a concern that the pandemic-induced stay-at-home orders and social restrictions may negatively affect weight reduction outcomes post-bariatric surgery. This study aimed to examine the impact of the COVID-19 on weight loss up to six months after three types of bariatric surgeries including One-Anastomosis Gastric Bypass (OAGB), RY Gastric Bypass (RYGB), and Sleeve Gastrectomy (SG) before and two time periods during the COVID-19 pandemic.

**Methods:**

We conducted a retrospective study using data from a comprehensive database of bariatric surgery patients in Iran. We recruited 882 patients who underwent bariatric surgery from the initiation of COVID-19 to 6 months before public vaccination (Time period 3); among them, 311 patients underwent surgery in the first six months of the pandemic (Time period 2). These patients were compared with 1368 ones in the control group who completed their 6 months follow-up before the pandemic. The study compared the BMI reduction, excess weight loss (EWL), and total weight loss (TWL) outcomes between these groups using Generalized Estimating Equations (GEE) with gamma distribution to adjust for factors that were unevenly distributed across the groups.

**Results:**

The age of participants in time periods 2 and 3 had a mean (standard deviation) of 38.97 (10.99) and 38.84 (10.71), respectively. In all groups, the majority of patients were females, accounting for 76.02%, 71.06%, and 75.74% for the control group and time periods 2 and 3, respectively. There was no significant difference between the groups in terms of weight reduction, as measured by BMI reduction, EWL, and TWL (related *P* values: 0.283, 0.465 and 0.169). Regression analysis indicated that higher baseline BMI values were associated with greater BMI reduction (0.04, 95% CI: 0.03–0.05), but this did not translate to higher EWL or TWL. Males showed greater BMI reduction (0.33, 95% CI: 0.18–0.49) and EWL (1.58, 95% CI: 0.79–2.37) than females, while females achieved higher TWL compared to males. Among different types of surgery, the OAGB resulted in more weight reduction among patients in the study.

**Conclusion:**

In conclusion, our study reveals that bariatric surgery remains effective for weight reduction during the first 6 months of the pandemic in Iran. Implementation of pandemic protocols ensures comparable efficacy to non-pandemic times. However, caution is needed in generalizing results beyond our specific context due to study limitations. Further research is essential to comprehensively assess the pandemic's broader impact on bariatric surgery outcomes under varying conditions.

## Background

The outbreak of the COVID-19 pandemic was a serious worldwide health issue [1]. Additionally, it changed most health services and at its peak, bariatric surgery has come to a halt. For the bariatric community, it would be concerning if bariatric surgery failed to achieve its expected results because of the risk of the stay-at-home requirement implemented in many cities, which often resulted in considerable alterations to daily routines and difficulties in weight management during the introduction of social restrictions [2, 3]. Evidence has shown that COVID-19-related restrictions promote unhealthy eating patterns, increase sedentary behavior, and delay medical care [4], which could affect the patient’s weight reduction who operated for bariatric surgery [5, 6].

On 20 February 2020, the Iranian government publicly announced the observation of COVID-19 cases in Iran, and to reduce the risk of spreading the virus, imposed restrictions such as social distancing and closing public and educational areas like gyms, sports facilities, and schools in all regions [7]. These constraints on public health probably limit possibilities for physical activity. For instance, before the pandemic, a research study revealed that 33% of Iranian adults did not meet the recommended levels of physical activity [8]. However, a recent survey conducted during COVID-19 in Iran showed that 78% of participants failed to reach the recommended physical activity levels [9].

Another aspect of the pandemic could be changing dietary behaviour. Individuals' dietary habits can be negatively influenced by the increased stress associated with the COVID-19 pandemic, particularly during the lockdown period. This may result in emotional eating behaviours and increased snacking [10, 11]. On the other hand, the pandemic may have also resulted in healthy eating habits, such as decreased fast food consumption, which may be related to the lack of food-associated social occasions and eating out [11].

Since the success of bariatric surgery is dependent on the progressive adoption of a healthy lifestyle and nutritional adjustments, it seems conceivable that the COVID-19 pandemic presented unique obstacles to weight loss in patients after bariatric surgery [12]. Some research has been done to document how the COVID-19 lockdown affects weight reduction after bariatric surgery. However, given that COVID-19 is a new situation, its impact may vary across different societies [13]. Moreover, variations in the duration and conditions of lockdown adherence contributed to the degree that it impacts the lifestyle changes of the patients [14, 15].

This paper presents a pioneering study that aims to investigate the influence of the COVID-19 pandemic on weight reduction outcomes after bariatric surgery among Iranian patients. Our database, which encompasses a wide range of patient data, allows us to explore this unique research question with a level of depth and accuracy not previously achieved [16, 17].

Through our meticulous analysis, we seek to uncover valuable insights that will contribute to the existing body of knowledge in the field of bariatric surgery, particularly in relation to the effects of the pandemic. By addressing this crucial research gap, our paper aims to provide valuable information and enhance our understanding of the interplay between the COVID-19 pandemic and weight reduction outcomes post-bariatric surgery in the Iranian population. The findings of this study are expected to serve as a foundation for future research, ultimately leading to improved patient care and informed decision-making in the field of bariatric surgery.

## Materials and methods

In this study, we have defined two specific time intervals based on significant milestones related to the pandemic in Iran. The first interval is defined from the initial confirmation of the first COVID-19 case in Iran, which occurred on 20th February 2020. Patients who underwent bariatric surgery from this date until six months later, specifically until 20th August 2020, were included in the first group of our study. This period represents the early phase of the pandemic in Iran.

The second interval starts on the same date, 20th February 2020, but extends until six months before the point when the public vaccination coverage reached 5% in the country. This milestone occurred on 13th February 2021. Patients who underwent bariatric surgery during this period are included in the second group. This interval allows us to examine the impact of the pandemic during a more advanced phase when vaccination efforts were underway.

For comparison, we established a control group comprising patients who underwent surgery two years before the emergence of COVID-19 in Iran. Specifically, patients who had their surgery on or before 20th February 2018 were included in this group. Their six-month follow-up period concluded before the onset of the pandemic.

The study's inclusion criteria comprised individuals who had undergone surgery within specific intervals, completed a six-month follow-up, and received one of the following procedures: OAGB, RYGB, or SG. Exclusions from the study encompassed patients who underwent a secondary operation, were infected with symptomatic COVID-19, or were pregnant.

**Fig. 1:**
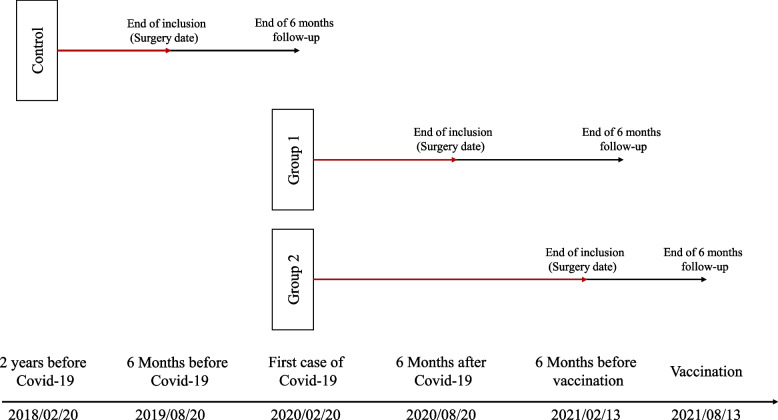
Study design according to eligible entry date (surgery date) and follow-up date

The study design, including the time of entry and group assignments, is depicted in Fig. 1. This diagram provides a visual representation of the patient selection process and illustrates how individuals were assigned to the respective study groups based on their surgery dates and adherence to the exclusion criteria. The figure helps to illustrate the robustness and integrity of our study design, ensuring that only eligible patients were included in the final analysis.

We measured patients' weight on the surgery day and follow-up visits at ten days, 1, 3, and 6 months after surgery. If a patient missed a follow-up visit, we made a phone call to gather the data and medical history. The patient's height on the surgery date was the reference value in the BMI calculation. In addition, medical documents made available demographical variables (age at surgery and sex) and comorbidity status (type 2 diabetes, hypertension, and hypothyroidism).

In each visit after the COVID-19 pandemic or phone calls interview, we asked the patients for their confirmed history of COVID-19. In this regard, we were able to capture moderate to severe infectious which may affect the weight reduction process. In addition, we sent a text message to all eligible patients and asked them to report their confirmed history of COVID-19 infection using the online questionnaire.

Furthermore, we took several precautions to ensure the safety of our patients and surgical team during the COVID-19 pandemic. Firstly, we followed the recommendations provided by the Society of American Gastrointestinal and Endoscopic Surgeons (SAGES) and The European Association for Endoscopic Surgeons (EAES) regarding metabolic and bariatric surgical practice during the pandemic. Additionally, we implemented a pre-operative screening protocol for COVID-19 to identify any patients who may have been infected and postponed their surgery until they were no longer infectious [18].

### Statistical methods

The baseline characteristics of patients were described using the frequency (proportion) and mean (standard deviation) for categorical and continuous variables, respectively. The baseline covariates of patients in group 1 (first period) and group 2 (second period) were compared with the control groups using an independent T-test and Chi-squared test for categorical and continuous variables. The significant level for all tests was set at < 0.05.

The measures of weight reduction were calculated as follows:

Excess Weight Loss (EWL) is a measure used to quantify the amount of weight loss achieved by a patient after undergoing weight loss surgery, relative to their excess weight. It is typically expressed as a percentage. The formula to calculate EWL is as follows:

$$\mathrm{EWL }\left(\mathrm{\%}\right)= \left[\left(\mathrm{Initial\,Weight}-\mathrm{Current\,Weightt}\right) / \left(\mathrm{Initial\,Weight}-\mathrm{Ideal\,Weight}\right)\right]\mathrm{ x }\,100$$

Initial Weight is the patient's weight before undergoing weight loss surgery. Current Weight is the patient's weight at the time of measurement. Ideal Weight is the weight corresponding to a healthy BMI (Body Mass Index) for the patient's height.

Total Weight Loss (TWL) is a measure used to determine the overall weight reduction achieved by a patient after weight loss surgery. It is also expressed as a percentage. The formula to calculate TWL is as follows:

$$\mathrm{TWL }\left(\mathrm{\%}\right)= \left[\left(\mathrm{Initial\,Weight}-\mathrm{Current\,Weight}\right) /\mathrm{ Initial\,Weight}\right]\mathrm{ x }\,100$$

The outcome variables of this study are BMI reduction, EWL and TWL. As a Shapiro–Wilk test did not confirm these outcomes follow the normal distribution, therefore we conducted a Generalized estimating equation (GEE) model using Gamma distribution to adjust the factors that were differently distributed across groups to achieve a more precise effect of groups. The model was fitted using identity link function and exchangeable covariance function between visit times.

## Results

The baseline characteristics of patients in the study are presented in Table 1. We recruited 882 patients in group 2 (from initiation of COVID-19 to 6 months before public vaccination); among them, 311 cases underwent surgery in the first six months of the pandemic (group 1). These patients were compared with 1368 cases in the control group. Patients' initial BMI in pandemic periods was significantly (*P* value < 0.001) lower than in the control group. The mean BMI for the control group was 45.13 kg/m^2^ compared to 43.82 kg/m^2^ in group 1 and 43.47 kg/m^2^ in group 2. The mean age of patients was 41.40 years in the control group compared to 38.84 in group 2 (*P* value < 0.001) and 38.97 in group 1(*P* value < 0.001). The RYGB surgery proportion was higher in patients before the pandemic, with a value of 25.29%. On the other hand, less than 2 percent of patients underwent RYGB after the pandemic.

**Table 1:** Baseline characteristics of patients in the study by their group allocation

**Variables**	**Control** **(**1368**)**	**Group 1** **(**311**)**	**Group 2** **(**882**)**	***P* value**^**1**^	***P* value**^**2**^
BMI before surgery (kg/m^2^)	**45.13 (6.10)**	**43.82 (5.67)**	**43.47 (5.67)**	** < 0.001**	** < 0.001**
Age at surgery (yrs.)	**41.40 (11.26)**	**38.97 (10.99)**	**38.84 (10.71)**	** < 0.001**	** < 0.001**
Sex, n (%)				0.068	0.877
Male	328 (23.98)	90 (28.94)	214 (24.26)		
Female	1040 (76.02)	221 (71.06)	668 (75.74)		
Hypothyroidism, n (%)	11 (2.09)	3 (3.03)	12 (3.23)	0.339	0.287
Diabetes, n (%)	11 (2.09)	2 (2.02)	18 (4.84)	0.966	0.021
Hypertension, n (%)	9 (1.71)	2 (2.02)	5 (1.34)	0.828	0.664
Surgery Type, M (SD)				** < 0.001**	** < 0.001**
SG	**441 (32.24)**	**149 (47.91)**	**410 (46.49)**		
OAGB	**581 (42.47)**	**156 (50.16)**	**457 (51.81)**		
RYGB	**346 (25.29)**	**6 (1.93)**	15 **1.70)**		

*M* Mean, *SD* Standard deviation, *OAGB* One-Anastomosis Gastric Bypass, *RYGB* RY Gastric Bypass, *SG* Sleeve Gastrectomy

^1^Comparing group 1 with control

^2^Comparing group 2 with control

**Fig. 2:**
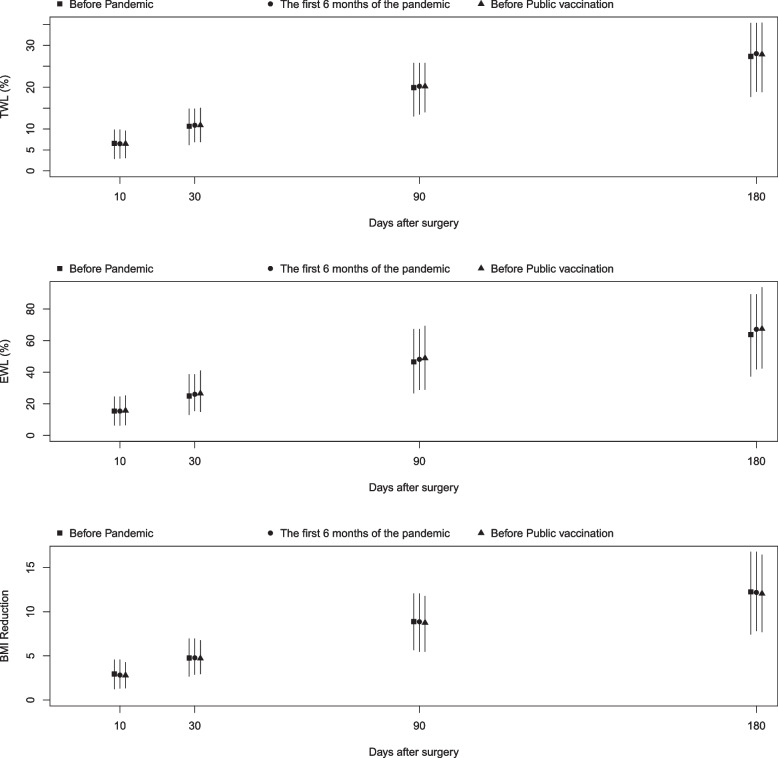
Trend of BMI reduction, EWL (excess weight loss) and TWL (total weight loss) in 6 month post bariatric surgery across time in different groups (before Pandemic, the first 6 months of the pandemic, before public vaccination)

In Fig. 2, we present the BMI reduction, EWL, and TWL along with their corresponding 95% confidence intervals for patients in all three groups. Our initial analysis indicated no significant difference between the groups in terms of weight reduction, as measured by all three metrics. However, to account for any potential confounding effects, we performed a regression analysis to adjust for other factors.

Table 2 presents the results of the GEE model, examining the weight reduction during the first 6 months of the pandemic compared to pre-pandemic times. The findings indicate that individuals with higher baseline BMI experienced greater BMI reduction (Coefficient: 0.04, 95% CI: 0.03, 0.05). However, this does not translate to higher EWL or TWL. Patients with higher BMI achieved less EWL (Coefficient: -0.31, 95% CI: -0.36, -0.27) and TWL (Coefficient: -0.03, 95% CI: -0.05, -0.01). Over the course of 10 days, the patients lost an average of approximately 0.62 BMI, 3.20% of EWL, and 1.40% of TWL. There was no statistically significant difference between patients who underwent surgery before the pandemic and those who underwent surgery within the first 6 months of the pandemic. Additionally, males experienced greater BMI reduction and EWL than females, while females achieved higher TWL compared to males.

**Table 2:** Comparison of GEE regression model results: surgery before pandemic vs. first 6 months of pandemic

Variables	**BMI reduction**		**EWL**		**TWL**	
	Coefficient (95% CI)	*P* value	Coefficient (95% CI)	*P* value	Coefficient (95% CI)	*P* value
Baseline BMI	**0.04** **(0.03, 0.05)**	** < 0.001**	**-0.31** **(-0.36, -0.27)**	** < 0.001**	**-0.03** **(-0.05, -0.01)**	**0.006**
Age at surgery (10 yrs.)	**-0.08** **(-0.14, -0.04)**	**0.001**	-0.19 (-0.47, 0.08)	0.173	**-0.22** **(-0.33, -0.11)**	** < 0.001**
Time (10 Days)	**0.62** **(0.61, 0.63)**	** < 0.001**	**3.20** **(3.13, 3.27)**	** < 0.001**	**1.40** **(1.37, 1.43)**	** < 0.001**
Period (Pandemic)	0.08 (-0.07, 0.24)	0.283	0.33 (-0.55, 1.20)	0.465	0.24 (-0.10, 0.58)	0.169
Hypothyroidism	0.011 (-0.27, 0.49)	0.577	0.09 (-1.93, 2.12)	0.928	0.31 (-0.51, 1.14)	0.457
Diabetes	0.19 (-0.19, 0.57)	0.319	-0.95 (-1.47, 3.38)	0.440	0.54 (-0.33, 1.42)	0.222
Hypertension	0.27 (-0.20, 0.73)	0.258	1.18 (-1.26, 3.61)	0.344	0.58 (-0.41, 1.57)	0.250
Sex (Male)	**0.33** **(0.18, 0.49)**	** < 0.001**	**1.58** **(0.79, 2.37)**	** < 0.001**	**-0.67** **(0.35, 0.99)**	** < 0.001**
Surgery Type (SG)						
OAGB	-0.09 (-0.21, 0.04)	0.180	-0.36 (-1.04, 0.33)	0.307	-0.22 (-0.49, 0.05)	0.117
RYGB	0.12 (-0.03, 0.28)	0.126	0.50 (-0.45, 1.45)	0.303	0.25 (-0.11, 0.60)	0.174

Estimates from Generalized Estimating Equation (GEE) from the Gamma family with identity link and robust standard errors, Total weight loss (TWL), Excess weight loss (Excess weight loss), *OAGB One*-Anastomosis Gastric Bypass, *RYGB* RY Gastric Bypass, *SG* Sleeve Gastrectomy

Table 3 presents the results of the GEE model, which assesses the weight reduction during the pandemic period before public vaccination compared to pre-pandemic times. The baseline BMI had a significant impact on BMI reduction, with higher initial BMI values associated with greater reduction (Coefficient: 0.04, 95% CI: 0.03, 0.05). However, this relationship did not translate to higher EWL or TWL. Patients with higher BMI experienced lower EWL (Coefficient: -0.35, 95% CI: -0.39, -0.31) and TWL (Coefficient: -0.03, 95% CI: -0.05, -0.02). During the study, this particular group of patients lost approximately 0.62 BMI units, 3.27% EWL, and 1.41% TWL every 10 days. There were no statistically significant differences observed in terms of BMI reduction and EWL between patients who underwent surgery before the pandemic and those who had surgery within the first six months of the pandemic. However, patients in the pandemic period experienced a slightly higher TWL reduction of 0.21%. In terms of gender differences, males demonstrated greater BMI reduction and EWL compared to females, although their TWL was lower than that of females.

**Table 3:** Comparison of GEE regression model results: surgery before pandemic vs. starting pandemic to before vaccination

Variables	**BMI reduction**		**EWL**		**TWL**	
	Coefficient (95% CI)	*P* value	Coefficient (95% CI)	*P* value	Coefficient (95% CI)	*P* value
Baseline BMI	**0.041** **(0.033, 0.05)**	** < 0.001**	**-0.35** **(-0.39, -0.31)**	** < 0.001**	**-0.03** **(-0.05, -0.02)**	** < 0.001**
Age at surgery (10 yrs.)	**-0.08** **(-0.12, -0.04)**	** < 0.001**	**-0.23** **(-0.05,0)**	**0.045**	**-0.2** **(-0.30, -0.11)**	** < 0.001**
Time (10 Days)	**0.62** **(0.61, 0.63)**	** < 0.001**	**3.27** **(3.21, 3.33)**	** < 0.001**	**1.41** **(1.38, 1.43)**	** < 0.001**
Period (Pandemic)	0.06 (-0.04, 0.15)	0.217	0.24 (-0.30, 0.89)	0.384	**0.21** **(0, 0.43)**	**0.047**
Hypothyroidism	0.11 (-0.17, 0.39)	0.440	0.02 (-1.58, 1.63)	0.976	0.20 (-0.42, 0.83)	0.520
Diabetes	0.29 (0.02, 0.56)	0.032	2.09 (0.45, 3.73)	0.013	0.65 (0.05, 1.25)	0.034
Hypertension	0.29 (-0.11, 0.69)	0.153	1.16 (-0.93, 3.25)	0.276	0.62 (-0.22, 1.46)	0.150
Sex (Male)	**0.37** **(0.26, 0.49)**	** < 0.001**	**1.81** **(1.17, 2.46)**	** < 0.001**	**0.77** **(0.52, 1.03)**	** < 0.001**
Surgery Type (SG)						
OAGB	**-0.12** **(-0.22, 0.02)**	**0.014**	**-0.74** **(-1.30, -0.19)**	**0.009**	**-0.34** **(-0.56, -0.13)**	**0.002**
RYGB	0.12 (-0.02, 0.26)	0.096	1.16 (-0.93, 3.25)	0.721	0.18 (-0.14, 0.51)	0.270

Estimates from Generalized Estimating Equation (GEE) from the Gamma family with identity link and robust standard errors, Total weight loss (TWL), Excess weight loss (Excess weight loss), *OAGB* One-Anastomosis Gastric Bypass, *RYGB* RY Gastric Bypass, *SG* Sleeve Gastrectomy

## Discussion

In this study conducted in Iran, we aimed to investigate the effect of the COVID-19 pandemic on weight reduction after bariatric surgery and compare outcomes between patients who underwent surgery before the COVID-19 pandemic, those who underwent surgery in the first six months of the pandemic and those who had surgery before public vaccination. The study found that there was no significant difference in weight reduction between pre-pandemic and the two pandemic periods. This finding suggests that the COVID-19 pandemic, at least during the initial six-month period and before widespread vaccination coverage, did not significantly alter the effectiveness of bariatric surgery in terms of weight reduction outcomes.

The lack of a significant difference in weight reduction between the pre-pandemic and pandemic surgery groups may have several underlying explanations. Firstly, bariatric surgery is known for its profound and lasting effects on weight reduction, typically resulting in substantial weight loss regardless of external factors [19]. It is now generally known that, particularly in the short term, the efficacy of surgery in promoting weight reduction is mediated by pleiotropic hormonal pathways [20]. The surgical intervention itself, combined with postoperative care and lifestyle modifications, may have outweighed any potential impact of the pandemic on weight outcomes.

Secondly, it is possible that the healthcare system in Iran effectively managed and maintained the quality of care for bariatric surgery patients throughout the pandemic [21, 22]. Adherence to established protocols, including preoperative evaluations, surgical procedures, and postoperative monitoring, may have remained consistent both in-person and through telemedicine, thus minimizing any negative influence on weight reduction outcomes.

Another contributing factor could be the relatively short duration of the initial six-month period of the pandemic considered in our study. Although it has been suggested that the nadir of weight reduction was achieved in 18 months post-bariatric surgery, the most weight reduction is related to the first 6 months of this period [23]. Therefore, focusing on this interval is as important as the whole duration. It is plausible that a longer follow-up period or inclusion of subsequent phases of the pandemic could reveal different results.

Additionally, due to the interaction of the COVID-19 pandemic with various social, economic, and cultural structures [24], lifestyle changes vary across countries. In Iran, research shows that people started eating healthier during the pandemic by consuming more homemade meals and less fast food due to stay-at-home orders and restaurant closures [25, 26]. There was also a significant decrease in sports activities as sports facilities were closed [25]. However, it should be noted that the level of physical activity in Iran is generally lower than in other countries. The national monitoring of non-communicable disease risk factors in Iran revealed that 35% of the population (46% females) had a sedentary lifestyle [27]. Thus, it is likely that the impact of reduced physical activity alone may have a smaller effect compared to other factors, such as improved nutrition. Additionally, studies investigating the effects of high-volume physical activity interventions on weight loss after bariatric surgery, have shown mixed results. Some studies have failed to demonstrate a significant impact on weight loss outcomes in the short-term and long-term [28].

Another study revealed worse weight loss outcomes one year after surgery when post-pandemic patients were compared to pre-pandemic patients, but it was limited to only SG intervention in a European center [5]. Huang et al. [19] achieved similar findings to the results of our study for SG (*n* = 121) and RYGB (*n* = 28) at the 1-year follow-up. The results showed no significant difference in 1-year EWL between pre- and post-COVID SG patients (51.7% vs. 55.9%, *p* = 0.35) or between pre- and post-COVID RYGB patients (88.9% vs. 80.4%, *p* = 0.42). Different healthcare systems, resource allocation, and pandemic management strategies across countries may yield varying outcomes in terms of weight reduction after bariatric surgery during the pandemic.

It is worth noting that the distribution of BMI was skewed towards category 2 and 3 obesity in the control group. This could be attributed to medical professionals' caution in selecting lower-risk patients during the pandemic, as individuals with a BMI > 50 kg/m^2^ are at a higher risk of complications than those with a lower BMI. For instance, a study by Kakarla et al. reported a significantly increased length of stay and a greater rate of 30-day mortality (0.26% versus 0.07%, odds ratio [OR] 4.38, *P* = 0.0001) in patients with higher BMI [29]. Alternatively, patients with higher motivation and fewer concerns may have been referred for surgery.

Finally, it should be highlighted that genetic and physiological variables may affect weight loss following surgery [30]. Nevertheless, it shouldn't be inferred that improving one's lifestyle following surgery is insignificant. The present design of obesity programs that rely on regular involvement in dietary advice, psychological support, and exercise routines is crucial. These qualities facilitate long-term and durable lifestyle modifications in patients, which promote sustained weight reduction [31].

It is crucial to acknowledge the limitations of our study. First, the focus on a specific geographic region may limit the generalizability of our findings. Further multicenter studies with larger sample sizes are warranted to strengthen the evidence base. Second, the short-term nature of our follow-up period restricts our understanding of long-term weight reduction outcomes and potential variations over time.

Additionally, there was little information available at the time on the patient's dietary habits, physical activity, and stress levels. In addition, the pandemic impacted patients differently and to varying degrees, which might result in heterogeneous data collection [4]. In addition, the pandemic effect on lifestyle changes over time and expanding this interval may bias the results. This study's strengths include large sample size, a variety of surgical procedures, and regular measures of weight following surgery.

## Conclusion

In conclusion, our study demonstrates that bariatric surgery's effectiveness, specifically in terms of weight reduction, remains consistent during the first 6 months in Iran, despite the pandemic situation. Adhering to pandemic protocols is essential for maintaining efficacy, comparable to regular non-pandemic times. However, it is important to acknowledge the limitations of our study for generalization beyond our specific context. Further research is warranted to comprehensively assess the broader impact of the pandemic on bariatric surgery outcomes under varying circumstances.

## Data Availability

Data supporting the conclusion of this article are not publicly available due to patient confidentiality rules in the Minimally Invasive Surgery Research Center (MISRC) but are available upon reasonable request, e.g., use of data by academic researchers, from the corresponding author (mazaherinezhad@yahoo.com) or Dr Somayeh Mokhber, the responsible officer at the MISRC (dr_so.mokhber@yahoo.com), for example, use of data by academic researchers.
